# Steady-state auditory evoked responses in audiological diagnosis in children: a comparison with brainstem evoked auditory responses

**DOI:** 10.1590/S1808-86942010000100016

**Published:** 2015-10-17

**Authors:** Gabriela Ribeiro Ivo Rodrigues, Doris Ruth Lewis, Silvia Nápole Fichino

**Affiliations:** 1 MSc in Speech and Hearing Therapy - PUC-SP, Speech and Hearing Therapist - Centro de Referência em Saúde Auditiva - Centro “Audição na Criança” - CeAC/ DERDIC/PUCSP; 2 PhD in Public Health - USP; Full Professor of the Graduate Program in Speech and Hearing Therapy - Pontifícia Universidade Católica de São Paulo - PUC/SP; Speech and Hearing Therapist - Centro de Referência em Saúde Auditiva - Centro “Audição na Criança” - CeAC/ DERDIC/PUCSP; 3 MSc in Speech and Hearing Therapy - PUC-SP, Speech and Hearing Therapist - Centro de Referência em Saúde Auditiva - Centro “Audição na Criança” - CeAC/DERDIC/PUCSP

**Keywords:** brain stem, auditory, evoked potentials

## Abstract

Auditory Steady-State Responses (ASSR) are being recognized as a promising technique in the assessment of hearing in children.

**Aim:**

To investigate the agreement level between results obtained from ASSR and click-ABR in a group of children with sensorineural hearing loss, in order to study the clinical applicability of this technique to evaluate the hearing status in young children.

**Study design:**

clinical prospective with a cross-sectional cohort.

**Materials and Methods:**

15 children aged between two and 36 months and with diagnosis of sensorineural hearing loss. The correlation between the responses of the two tests was evaluated by intraclass correlation coefficient and McNemar test comparing the probability of responses in both tests.

**Results:**

The correlation coefficients were: 0.70; 0.64; 0.49; 0.69; 0.63 and 0.68 respectively for frequencies of 1, 2, 4, 1–2, 2–4 and 1–2-4kHz. In McNemar test p = 0.000, indicating that the probability of obtaining responses in both tests was not equal, but greater for the ASSR.

**Conclusion:**

we found good agreement between the techniques among the four frequencies evaluated, suggesting that both tests may be complementary. However, the ASSR was able to obtain additional information in cases of severe and profound hearing losses, adding important data to the management of these children, providing greater accuracy to the audiological diagnosis.

## INTRODUCTION

With the neonatal hearing screening, aiming at improving the negative aspects associated with congenital hearing loss, there is the need to do an accurate diagnosis of the child's hearing status, in order to start to fit hearing aids and proceed with the necessary interventions. Studies show that the proper sound amplification followed by speech and hearing therapy in the first six months of life maximizes speech and hearing development potential in children with all levels of hearing loss.^1^,^2^

Nonetheless, to establish the audiometric profile in children in their first months of life is a rather complex task; having seen that it is not possible to obtain reliable results which depend on the behavioral responses from these patients. In this population, the audiologic diagnosis is then based on electroacoustics tests and, mainly, electrophysiological - which assess the integrity of the auditory pathways, enabling an estimate of the child's hearing.^3^

It is with such aim that the Brainstem Evoked Response Audiometry (BERA) obtained from the click stimuli (Click BERA) has been broadly used in recent decades. The click is considered more efficient to evoke electrophysiological responses since it stimulates a larger region of the cochlea, resulting in a good neural synchrony through the joint action of a larger number of nervous fibers. Nonetheless, since it is a broad band stimulus, the click is not frequency specific and has its concentration on the higher frequencies (2 to 4 kHz), in such as way that it does not provide information on the hearing loss configuration.^4^

In the clinical setting, the specific frequency brainstem evoked potentials (SF-BERA) has been used to estimate the hearing loss configuration. In such technique, the study is done as it is with the click-BERA; however the stimuli used are tonebursts, usually in the frequencies of 0.5, 1, 2 and 4 kHz; or even, 0.5, 1.5 and 4 kHz. Each frequency is studied alone and unilaterally, which extends considerably the test duration.^4^

The Stable State Evoked Auditory Potentials (SSEAP) brought about the promise of solving some of the click-BERA and SF-BERA limitations. The use of specific stimuli, introduced simultaneously, enables the assessment of four frequencies at the same time, making the recording of these potentials faster and more specific. The response detection provided by statistical methods brings about a reduction in the risks created by the subjective interpretation; and also the possibility of studying intensity thresholds stronger than the click-BERA and SF-BERA; also enabling the further assessment of the residual hearing in cases of profound hearing loss.^5^, ^6^, ^7^

Studies have compared BERA and click-BERA responses showing reasonable correlations among the techniques.^8^, ^9^, ^10^, ^11^, ^12^ The few studies comparing click-BERA and SSEAP in the frequencies of 2 and 4 kHz, and with the SSEAP mean values in the high frequencies (1–4 and 2–4 kHz), in children with ages equal to or lower than 36 months indicated significant correlations between the techniques.^10,^^11,^^13^

In this paper we will discuss the first clinical experiences with the SSEAP in a highly complex hearing health service in São Paulo. The experimental inclusion of the SSEAP among the electrophysiological tests enabled the comparison with the protocol previously established, in other words, the click-BERA. The present study aimed at investigating the level of agreement among the SSEAP and click-BERA tests in a group of children with sensorineural hearing loss, thus studying the clinical applicability of this technique in the audiological evaluation of children.

## MATERIALS AND METHODS

This study was held at the “Audição na Criança” Center – CeAC, serviço da DERDIC – Studies and Rehabilitation of Communication Disorders Division Pontifícia Universidade Católica de São Paulo - PUC/SP, and was approved by the Ethics Committee (protocol # 113/2008). All the guardians of the subjects of the study signed the “Free and Informed Consent Form”, thus consenting with the study and the disclosure of the results, according to Resolution 196/96.

We had 15 children with sensorineural hearing loss in the age range between 02 and 36 months (mean value of 17 months) enrolled in the study, adding up to a total of 30 ears. In order to confirm the hearing loss, we held the following procedures, according to the protocol established by the institution's diagnostic team: otolaryngological exam, behavioral audiologic evaluation, immittance measures, transient stimulus and distortion product otoacoustic emission recordings and electrophysiological tests (click-BERA and SSEAP) at the end of the tests, a new otolaryngological consultation is carried out in order to conclude each case studied.

The equipment used was the “SmartEP”, from Intelligent Hearing Systems (IHS). The tests were held under natural sleep, and the reference electrodes were placed on the right (A2) and left (A1) mastoids, and the active (Fz) and ground (Fpz) electrodes on the forehead.

### Click-stimulus Brainstem Evoked Response Audiometry (click-BERA)

In order to record the click-BERA we used the rarefaction polarity clicks with 100 μs duration in the repetition rate of 27.7/s. The analysis window was of 20 ms and filters were those of 100 and 3000 Hz. The responses were recorded at 10 dB steps and the maximum intensity used was 90 dBnHL. The criterion used to establish the presence of the response was the visual identification of wave V and its reproducibility.

### Steady State Evoked Auditory Potentials (SSEAP)

#### Stimulus

Each stimulus used was made up of the simultaneous combination of 4 tonepipes with the frequencies of 0.5, 1, 2 and 4 kHz, in the modulation frequencies of approximately 77, 85, 93 and 101 Hz on the left ear and of 79, 87, 95 and 103 Hz on the right ear, respectively.

#### Recording

We studied the minimum response level (MRL) found for the frequencies of 0.5, 1, 2 and 4 kHz simultaneously in both ears. The MRL were studied at 10 dB steps. As the responses were observed, with electrical noise below 0.05 μV, the test was interrupted and these frequencies were removed. The test in the remaining frequencies was then restarted, in the same intensity. Each simultaneous stimulus was presented bilaterally through ER-3A insertion phones. When it was not possible to do the bilateral test, it was done unilaterally. The initial intensity was deemed audible by the subject based on the behavioral evaluation and did not go beyond 110 dBSPL.

#### Analysis

The maximum number of stimuli was adjusted for 400 with 1.024s duration each, broken down in 20 scans. The EEG samples after the scanning were filtered with 30–3000 Hz filters, and amplified with a 1,000.0 K gain, and were then processed using an A/D 20 kHz conversion rate. After each scanning, the Fourier Fast Transformation (FFT) was automatically carried out by the software, showing the result obtained in a polar batch and in a frequency spectrum. The F test calculated that the response amplitude likelihood would be significantly different from the modulation frequency background mean amplitude, as well as the background noise mean amplitude in the adjacent frequencies. If the signal/noise ratio was higher than 6.13 dB (p = 0.05) in both conditions, the sign was considered a response.^14^,^15^

#### Results Conversion

The SSEAP results were transformed from dBSPL to dBHL according to the ISO 389-2 standard for insertion phones, with the corrections −6, −0, −3 and −6 dB respectively, for tones 0.5, 1, 2 and 4 kHz, a criterion already used in a study15 which used the same equipment.

#### Result analysis

The agreement between the SSEAP and click-BERA results was analyzed through the interclass correlation coefficient^16^. Both tests were compared one to the other as to the likelihood of response occurrence. For that, we built tables with the frequency distributions and joint percentages of these tests, and the likelihood of response occurrence compared by means of the McNemar test.^17^ The entire analysis was made by frequency, and in the hypothesis test the significance level was fixed in 5%.

## RESULTS

The click-BERA responses were compared to the SSEAP ones in the frequencies of 1, 2 and 4 kHz; and also with the mean values of 1–2, 1–4 and 1–2-4 kHz. The frequencies of 1, 2 and 4 kHz of the SSEAP were selected because the click-Bera result, in many cases, can correspond to the best response between 1 and 4 kHz.^18^

We carried out twelve comparisons between the pairs. The interclass correlation coefficients found between the SSEAP and the click-BERA are presented on Table 1.

**Table 1:** Interclass correlation coefficients observed between the click-BERA (dBnHL) and the SSEAP (dBHL).

Frequency (kHz)	Coefficient
1	0,70
2	0,64
4	0,49
1 e 2	0,69
2 e 4	0,63
1, 2 e 4	0,68

When the two tests were compared in terms of their response likelihood, the SSEAP presented a higher probability than the click-BERA. Results are shown on Table 2.

**Table 2:** Frequency distribution and joint percentages of SSEAP and click-BERA responses in the frequencies of 1,000 Hz, 2,000 Hz and 4,000 Hz.

1000 Hz			

BERA	SSEAP		Total
	Absent	Present	
Absent	1	17	18
	3,30%	56,70%	60,00%
Present		12	12
		40,00%	40,00%
Total	1	29	30
	3,30%	96,70%	100,00%
p= 0,000			
2000 Hz			

BERA	SSEAP		Total
	Absent	Present	
Absent	4	14	18
	13,30%	46,70%	60,00%
Present		12	12
		40,00%	40,00%
Total	4	26	30
	13,30%	86,70%	100,00%
p= 0,000			
2000 Hz			

BERA	SSEAP		Total
	Absent	Present	
Absent	4	14	18
	13,30%	46,70%	60,00%
Present		12	12
		40,00%	40,00%
Total	4	26	30
	13,30%	86,70%	100,00%
p= 0,000			

In order to exemplify the interpretation of this table, we considered the 1 kHz frequence. Of the 30 ears, one (3.3%) did not present an answer in the SSEAP and in the click-BERA; seventeen (56.7%) presented an answer in the SSEAP and no answer in the click-BERA, and twelve (40%), a response present in both tests. We observed the table marginal values, in the click-BERA the response was absent in eighteen ears (60%) and present in twelve (40%); in the SSEAP the response was absent in one ear (3.3%) and present in 27 (96.7%).

Below each session in the table we have the p-value obtained in the McNemar test, which compares the probabilities of presence in the two tests, considering that both are applied to the same ears. In the three frequencies we obtained a p=0.000, indicating that the likelihood of a response happening in the two tests is not the same, and the SSEAP probability was higher than in the click-BERA.

## DISCUSSION

The coefficients found point to a good agreement between the two techniques in the high frequencies, as already reported by studies which compared the click-BERA with SSEAP in the frequencies of 2 and 4 kHz;^8,^^11,^^13^ and by studies which included the frequency of 1 kHz in the comparison.^10^,^12^

The best correlation between click-BERA and SSEAP happened in the frequency of 1 kHz (0.70), followed by the mean value of 1–2 kHz (0.69) and the mean value of 1-2-4 kHz (0.68). The worst correlation was with the frequency of 4 kHz (0.49). These results are similar to the group with the sensorineural hearing loss.^12^

The coefficients found in previous studies varied between 0.77 and 0.958,^11^,^13^, being better than the ones obtained in the present study, which varied between 0.49 and 0.70. Notwithstanding, the coefficients obtained by Swanepoel and Ebrahim^12^ in the population with sensorineural hearing loss were lower than the ones obtained in this study, varying between 0.24 and 0.65.

The variability between the coefficients obtained in the different studies can be assigned to the different methodologies employed, as well as the hearing loss configurations which made up the samples. Since the click-BERA can correspond to the best response between 1 and 4 kHz, a sample made up of a larger number of hearing loss with descending configurations, for instance, may present a better correlation with the SSEAP for the frequency of 1 kHz.^12^,^18^

Swanepoel and Ebrahim^12^ found a better click-BERA and SSEAP for the 2–4 kHz frequencies in subjects with normal hearing and conductive hearing loss. Notwithstanding, in the subjects with sensorineural hearing loss, the best correlation was with the mean value of 1–4 kHz.

**Figure 1:**
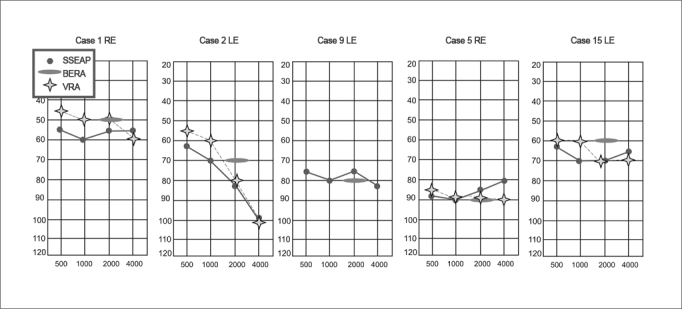
Examples of cases comparing the click-BERA and the SSEAP I. - SSEAP: Steady State Evoked Auditory Potentials; Click-BERA: Brainstem Evoked Response Audiometry with the click; VRA: Visual Reinforcement Audiometry.

In general, the click has its power concentrated between 2 and 4 kHz, indicating hearing loss for the high frequencies; however, in cases of descending hearing loss, its broad band nature may represent the low frequencies.^12^,^18^ Cases 1, 2 and 15, illustrated on Fig. 1, are typical examples of this relationship.

It is still worth stressing that, although the click-BERA is broadly used in the clinical routine in order to estimate hearing thresholds, its relation with the behavioral thresholds is not always exact. Studies from the 70′s and 80′s already reported correlations varying between 0.40 and 0.75 between the results of the click-BERA and the behavioral thresholds.^19^,^20^ Then, it is evident that the click-BERA can correlate well with the frequencies of 1, 2 and 4 kHz, but they do not accurately reflect a single region of the cochlea, and such relation is much variable.^21^

Cone-Wesson et al.^22^ showed strong correlations between the click-BERA and the SSEAP in the frequencies of 1, 2 and 4 kHz, and also in the frequency of 0.5 kHz (0.78), showing that the click-BERA can estimate residual hearing in any frequency between 0.5 and 4 kHz, and, therefore, be compared to the SSEAP in all frequencies.

When the SSEAP and the SF-BERA were compared as to the probability of response occurrence, the SSEAP presented a much higher probability. Studies have pointed to a SSEAP advantage in estimating the residual hearing in profound hearing loss which will not show responses in the click-BERA.^8,^^10,^^23^

**Figure 2:**
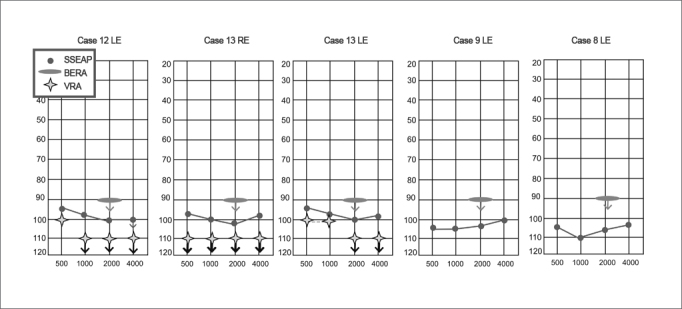
Examples of cases comparing the click-BERA and the SSEAP II. - SSEAP: Steady State Evoked Auditory Potentials; Click-BERA: Brainstem Evoked Response Audiometry with the click; VRA: Visual Reinforcement Audiometry.

In our findings, the SSEAP indicated a residual hearing in the absence of click-BERA recordings, as in the cases illustrated on Fig. 2.

It is a consensus that click-BERA recordings are limited to the study of strong intensities. The maximum intensity value available in most of the equipment is 90 to 100 dBnHL, which is not enough to properly measure the profound hearing loss.^23^

The continuous stimuli used in the SSEAP recordings enabled us to better study the stronger intensity hearing thresholds when compared to the click-BERA. Nonetheless, there are reports of artifacts and the supposed presence of vestibular responses when the SSEAP are presented in strong intensities.^24^,^25^

So far, there is no report of any artifact in strong intensities with the system used in this study. Nonetheless, because of the very impossibility of performing a VRA with the insertion phones in many cases, one can not rule out the likelihood of artifacts present, such as in cases 8 and 9 illustrated on Fig.2, in which the VRA was not carried out, or in cases 12 and 13, in which there are responses present in the SSEAP when VRA is not present.

It is clear, however, that the cases in which the click-BERA were absent, the SSEAP responses indicated better residual hearing, as in the cases illustrated on Fig.1.

The advantages of estimating hearing by frequency specificity and determining residual hearing in the cases of profound hearing loss shown at the SSEAP when compared to the click-BERA are well reported in the literature.^8,^^10,^^11,^^23^

However, the click-BERA, also presented advantages which must be considered when one discusses the applicability of the tests in clinical practice, since it adds information which can not be obtained by means of the SSEAP. This information is related to the type of hearing loss, if conductive or sensorineural, and those necessary for the differential diagnosis of the hearing neuropathy.^13,^^15,^^26^

For this reason, we deemed important to use the click-BERA and the SSEAP together, in such a way that one completes and confirms the information of the other; by the same token, logoaudiometry is used to confirm pure tone thresholds, as suggested by Cone-Wesson et al.^22^

We must also stress that some studies have suggested that the decisions about referring a patient to cochlear implant are reinforced with the SSEAP, as well as the decision about which ear will be implanted.^8,^^10,^^23^

In fact, although the SSEAP were not routinely done in clinical practice, and even considering that this study is experimental, these potentials add important information. Interpreted together with the other procedures, they enabled the referral of five cases for cochlear implant. In all the cases, the VRA could not be carried out with insertion phones, which differentiates the hearing status of each ear separately.

## CONCLUSION

The comparison between the click-BERA and the SSEAP responses in the 1, 2 and 4 kHz frequencies in 15 children with sensorineural hearing loss led to the conclusion that there is a good agreement between the responses of both techniques in the frequencies evaluated. Moreover, the SSEAP brought about additional information in the cases of severe and profound hearing loss. The results obtained from the click-BERA, and the SSEAP recordings tend to add important hearing data, especially when it is not possible to obtain reliable behavioral responses - adding data to the battery of tests with children with hearing loss and providing for an audiological diagnosis and a more accurate hearing aid fitting.
